# Extract of Sheng-Mai-San Ameliorates Myocardial Ischemia-Induced Heart Failure by Modulating Ca^2+^-Calcineurin-Mediated Drp1 Signaling Pathways

**DOI:** 10.3390/ijms18091825

**Published:** 2017-08-25

**Authors:** Ye Yang, Yushan Tian, Siyao Hu, Suxia Bi, Suxia Li, Yuanjia Hu, Junping Kou, Jin Qi, Boyang Yu

**Affiliations:** 1 State Key Laboratory of Natural Medicines, Jiangsu Key Laboratory of Traditional Chinese Medicine Evaluation and Translational Research, Department of Complex Prescription of Traditional Chinese Medicine, China Pharmaceutical University, Nanjing 211198, China; yangye459@163.com (Y.Y.); yushan126163@126.com (Y.T.); hu_siyao@126.com (S.H.); suxiabi@163.com (S.B.); lsx0601@126.com (S.L.); junpingkou@cpu.edu.cn (J.K.); 2 State Key Laboratory of Quality Research in Chinese Medicine, Institute of Chinese Medical Sciences, University of Macau, Macau 999078, China; yuanjiahu@umac.mo

**Keywords:** extract of Sheng-Mai-San, heart failure, calcineurin A, dynamin-related protein 1, mitochondrial fission

## Abstract

Sheng-Mai-San (SMS) is a well-known traditional Chinese medicine (TCM) complex prescription used to treat heart failure (HF) and angina in clinic. However, its potential therapeutic mechanisms remain unclear. The present study evaluated the cardioprotection of extract of SMS (ESMS) on myocardial ischemia (MI)-induced HF, and explored the underlying molecular mechanisms. The results demonstrated that ESMS (728.0 mg/kg) significantly attenuated MI injury-induced HF by improving cardiac function and pathological changes, decreasing lactate dehydrogenase (LDH), creatine kinase (CK) activities, and brain natriuretic peptide (BNP) levels; increasing ATPase activity; and reducing intracellular Ca^2+^ levels in MI-induced HF mice model. It also significantly decreased the apoptotic index. In vitro, ESMS (400 μg/mL) inhibited mitochondrial-dependent myocardial apoptosis by modulating the expression of caspase-3 and the Bcl-2/Bax ratio, and improved mitochondrial function through increasing mitochondrial membrane potential and cellular ATP content. ESMS restored intracellular Ca^2+^ and downregulated the expression of Calcineurin A (CnA), thus inhibiting phosphorylation of dynamin-related protein 1 (Drp1) at Ser616 and increasing phosphorylation of Drp1 at Ser637 to prevent cardiomyocyte mitochondrial fission. Above-mentioned results demonstrated ESMS suppressed mitochondrial-mediated apoptosis in oxygen glucose deprivation (OGD) injured H9c2 cardiomyocytes. These findings suggested that ESMS attenuated MI-induced HF by regulating Ca^2+^ homeostasis and suppressing mitochondrial mediated apoptosis through the modulation of Ca^2+^-calcineurin-mediated Drp1 signaling pathways. Our results provide insight into the mechanism and clinical applications of SMS and suggest a potential therapeutic strategy for HF.

## 1. Introduction

Heart failure (HF) is a complex clinical syndrome and is recognized as one of the most burdensome diseases in the world [1,2]. According to the American College of Cardiology Foundation/American Heart Association (ACCF/AHA) statistics, even though pharmacological and device-based treatments have improved survival, the five-year mortality rate of HF patient remains at an alarming 50%, and the search for better therapies is one of the major challenges in HF [3]. In recent years, accumulating evidence indicates that myocardial ischemia (MI) has become the most important cause of HF [4]. MI causes substantial left ventricular damage and induces acquired abnormalities in cardiac structure and function such that the heart ability to fill or eject blood is impaired; these changes may lead to the further deterioration followed by HF [5]. However, the mainstay of the current treatment is based on inhibitors of chronic neurohumoral activation in HF, such as statins, β-adrenergic blockers, and angiotensin converting enzyme (ACE) inhibitors [3,6]. These treatments are often limited to providing symptomatic relief and temporarily impeding disease progression [6]. Therefore, identifying potential cardioprotective agents and elucidating their underlying mechanisms is important to prevent ischemia induced HF.

Over the past centuries, traditional Chinese medicine (TCM) has been used to prevent or treat imbalances in the body. Scientific evidence supports the safety and efficacy of TCM for the treatment of specific ailments. Treatments consisting of multiple drugs directed against multiple pathways and targets have stronger therapeutic efficacy for complex multifactorial diseases [7,8]. The effective evidence from clinical trials contributes to the acceptance of TCM can be useful in the treatment of cardiovascular diseases [9,10,11].

Sheng-Mai-San (SMS) is a well-known TCM complex prescription composed of *Panax ginseng* C. A. Mey, *Ophiopogon japonicus* (Thunb.) Ker-Gawl., and *Schisandra chinensis* (Turcz.) Baill. (1:3:1.5). SMS is used for the treatment of palpitations, lassitude, and shortness of breath, which are symptoms of effort angina in clinical practice. Both clinical and basic studies show that SMS can be used for the prevention and treatment of cardio-cerebral ischemic diseases, it has shown better efficacy and fewer side effects than standard medical treatments [10,12]. Previous studies mainly explored the antioxidant capacity of SMS to protect against ischemic myocardial dysfunction [13,14,15]. In addition, the effects of SMS have been mostly investigated using isoproterenol-induced myocardial injury models or myocardial ischemia-reperfusion caused by coronary artery ligation [16,17]. Meanwhile, SMS can improve the tolerance to myocardial hypoxia, enhance myocardial systole, ameliorate left ventricular ejection fraction (LVEF), regulate blood vessel function, modulate immunity, and has anti-lipid peroxidation effects [11,18,19]. However, the potential molecular mechanisms of SMS and its activity against MI-induced HF remain to be explored.

Cardiac mitochondria are responsible for generating energy in the form of ATP through oxidative phosphorylation. Mitochondria are critical for cardiac function, structural and functional abnormalities of mitochondria contribute to several common cardiovascular diseases including HF [20,21]. Mitochondria are dynamic organelles that continuously moving, fusing, and dividing. The fusion and fission of mitochondria causes changes in mitochondrial shape [22]. Several recent studies indicated that increased mitochondrial fission is directly involved in cardiomyocyte death and dynamin-related protein 1 (Drp1), providing a driving force in fission, plays a pivotal role in MI injury [23]. Two major phosphorylation sites of Drp1 were reported. Phosphorylation of Drp1 at Ser616 results in mitochondrial fission, whereas phosphorylation at Ser637 blocks fission and induces fusion and elongation of mitochondria [24,25].

Evidence suggests that calcium ion (Ca^2+^) homeostasis is important for cellular homeostasis [26,27]. Ca^2+^, the second messenger in the heart, relates the muscle contraction and relaxation in the cardiac cycle to mitochondrial energy production [28,29]. In MI, cardiac contractility is impaired by the rhythmic release and reuptake of Ca^2+^ within the cardiomyocytes. Meanwhile, Ca^2+^ homeostasis is disrupted, leading to alterations of mitochondrial structure and function [30]. Previous studies showed that intracellular Ca^2+^ levels modulated the activation of calcineurin [31], and sustained calcineurin activation is sufficient to promote cardiac structural and functional injury [32,33]. In addition, it is the calcineurin activity that can cause increase mitochondrial fission involving the phosphorylation of Drp1. Drp1 is recruited to the mitochondrial membrane, which accelerates mitochondrial fission, resulting in the fragmentation of the mitochondrial network [25,31]. Therefore, regulating Ca^2+^ homeostasis and suppressing mitochondrial fission might be beneficial for the treatment of MI-induced cardiomyocyte injury.

In the current study, we tested the effects of extract of SMS (ESMS) in vivo on heart structural and functional impairment caused by permanent coronary artery ligation (CAL)-induced MI following HF, and in the H9c2 cardiomyocyte cell line subjected to oxygen glucose deprivation (OGD) injury in vitro. Then, we investigated the therapeutic effect of ESMS and explored its underlying mechanisms of ESMS. We found that ESMS attenuated MI injury-induced HF by modulated Ca^2+^-calcineurin-mediated Drp1 signaling pathways, regulated Ca^2+^ homeostasis, and suppressed mitochondrial mediated apoptosis. Our findings provide evidence supporting the clinical application of SMS and a potential therapeutic strategy for the treatment of HF.

## 2. Results

### 2.1. Extract of Sheng-Mai-San (ESMS) Ameliorated Cardiac Function and Decreased Myocardial Injury in Myocardial Ischemia (MI)-Induced Heart Failure (HF) Mice

**Figure 1:**
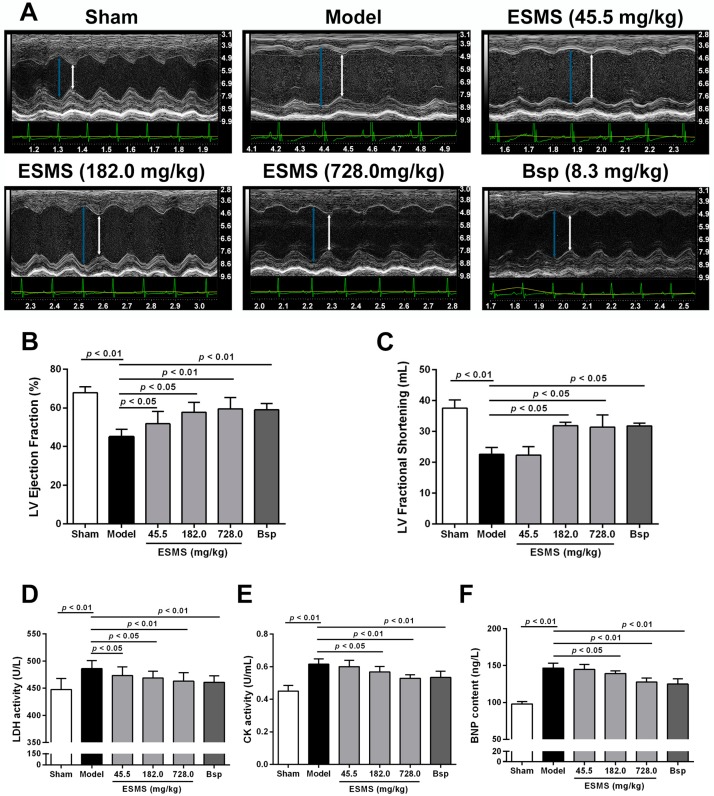
Extract of Sheng-Mai-San (ESMS) ameliorated cardiac function and decreased myocardial injury in myocardial ischemia (MI)-induced heart failure (HF) mice. (**A**) Representative M-mode of echocardiograms. Cardiac performance was determined by echocardiography after three weeks of coronary artery ligation (CAL) in mice. The green peaks show the real-time change in the heart rate of the mouse; the yellow peaks indicate the real-time change of the body temperature; the long blue arrow indicates cardiac diastole; and the short white arrow shows cardiac systole; (**B**) Left ventricular (LV) ejection fraction changes; (**C**) LV fractional shortening changes; (**D**) The release of: lactate dehydrogenase (LDH), (**E**) creatine kinase (CK), (**F**) brain natriuretic peptide (BNP) were determined in serum after three weeks of CAL in mice (*n* = 8). Results were presented as mean ± SD. Bsp = Bisoprolol (positive control).

Echocardiography was performed to determine the effects of ESMS on cardiac function. Representative images of M-mode echocardiograms are shown in Figure 1A. After three weeks of MI, LVEF and left ventricular fractional shortening (LVFS) were significantly reduced in the model group (Figure 1B,C), indicating reduced cardiac contractile function. Interventricular septum in diastole (IVS; d), left ventricle interior diameter in diastole (LVID; d), LV posterior wall in diastole (LVPW; d), LV volume in diastole (LV Vol; d), and LV Mass were significantly increased, whereas relative wall thickness (RWT) was significantly decreased (Figure S2A–F), indicating the development of pathological hypertrophy and cardiac remodeling. ESMS (728.0 mg/kg) and Bisoprolol (8.3 mg/kg) groups showed significant improvement. Bisoprolol, a β-adrenergic blocker widely used to treat heart failure, was used as a positive control. This data indicated that ESMS increased the contractile capability and prevented the development of pathological hypertrophy.

The levels of lactate dehydrogenase (LDH), creatine kinase (CK), and brain natriuretic peptide (BNP) in serum were evaluated to determine the effects of ESMS on myocardial damage. As shown in Figure 1D–F, LDH, CK, and BNP were significantly increased in the model group, which indicated that myocardial injury was aggravated and the symptoms of left ventricular (LV) dysfunction increased after three weeks of MI in mice. However, ESMS (728.0 mg/kg) and Bisoprolol (8.3 mg/kg) groups showed markedly decreased levels of LDH, CK, and BNP in serum, indicating that ESMS could decreased myocardial damage and the risk of HF.

### 2.2. ESMS Ameliorated Cardiac Pathological Changes and Fibrosis in MI-Induced HF Mice

**Figure 2:**
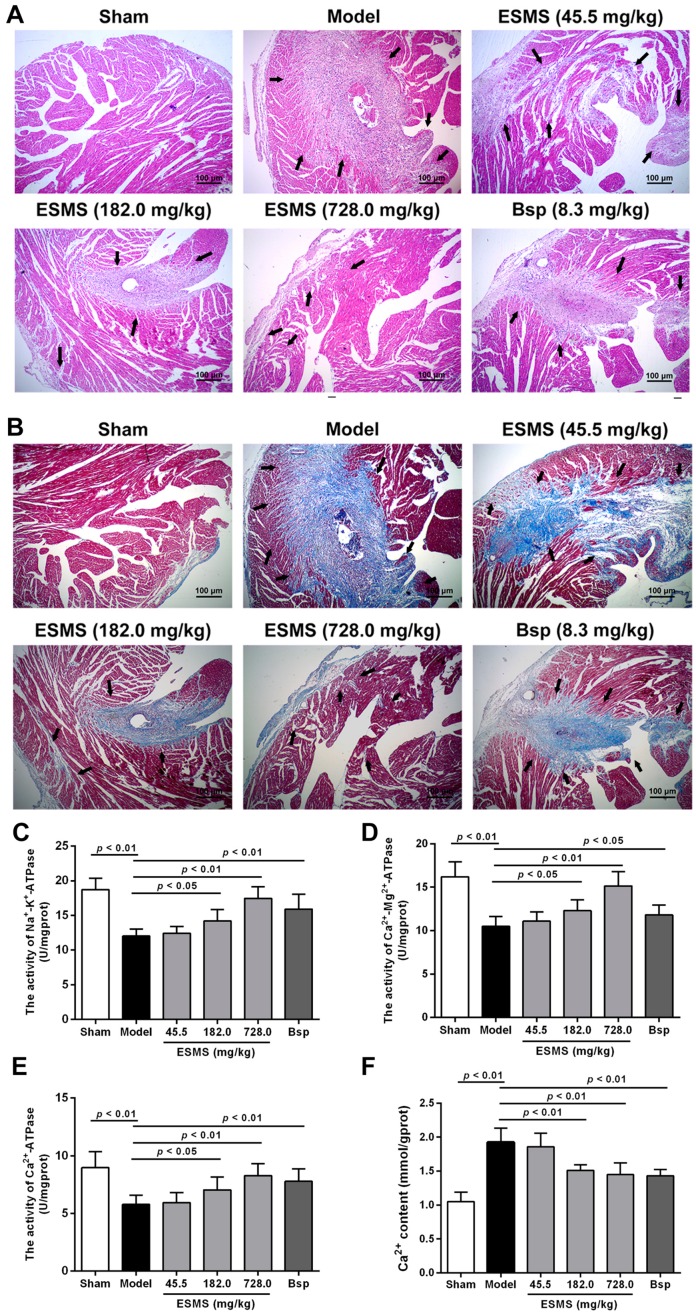
ESMS ameliorated cardiac pathological changes and fibrosis, increased ATPase activity and reduced intracellular Ca^2+^ levels in MI-induced HF mice. (**A**) Histopathological changes of representative myocardium sections were measured by H&E staining (200× magnification). The arrow indicates cardiac pathological changes. Bar is 100 μm (*n* = 6); (**B**) Myocardial fibrosis was measured by Masson staining (200× magnification). Left ventricular wall fibrosis (collagen staining in blue color) was increased, and the arrow indicates interstitial fibrosis. Bar is 100 μm (*n* = 6); (**C**–**E**) Na^+^-K^+^-ATPase, Ca^2+^-Mg^2+^-ATPase, Ca^2+^-ATPase activity was determined in tissue after three weeks of CAL in mice (*n* = 8); (**F**) Intracellular Ca^2+^ levels were determined in tissue after three weeks of CAL in mice (*n* = 8). Results were presented as mean ± SD. Bsp = Bisoprolol (positive control).

The pathological changes in the myocardium were examined by hematoxylin-eosin (H&E) staining of the heart. As shown in Figure 2A, at three weeks after MI induction, the data indicated severe morphological damages, including widespread myocardial structural disarray, the areas of necrosis and fusion increased, and a large number of inflammatory cells infiltrated the myocardial tissue in model mice. Assessment of fibrosis by Masson’s staining showed that cardiomyocytes were lysed or fractured, the myocardial structure was disordered, nuclei were absent, and fibroblasts had infiltrated into the heart in model mice (Figure 2B). As shown in Figure S3, the quantification of fibrotic areas was measured, ESMS treatment, especially at a dose of 728.0 mg/kg, restored the histopathological damage in the heart and reduced fibrosis, indicating that ESMS ameliorated cardiac pathological changes and protected against fibrosis.

### 2.3. ESMS Increased ATPase Activity and Reduced Intracellular Ca^2+^ Concentration in MI-Induced HF Mice

As shown in Figure 2C–E, during MI, Na^+^-K^+^-ATPase, Ca^2+^-Mg^2+^-ATPase, and Ca^2+^-ATPase activities were significantly decreased in the model group compared with those in the sham group, whereas the ESMS (728.0 mg/kg) significantly increased ATPase activity. Meanwhile, since elevation of intracellular Ca^2+^ levels could result in cardiac hypertrophic responses, we assessed whether the Ca^2+^ levels were affected. As shown in Figure 2F, during MI, intracellular Ca^2+^ concentration was significantly increased in the model group, whereas the ESMS (728.0 mg/kg) significantly reduced the intracellular Ca^2+^ levels.

### 2.4. ESMS Inhibited Myocardial Apoptosis in MI-Induced HF Mice

**Figure 3:**
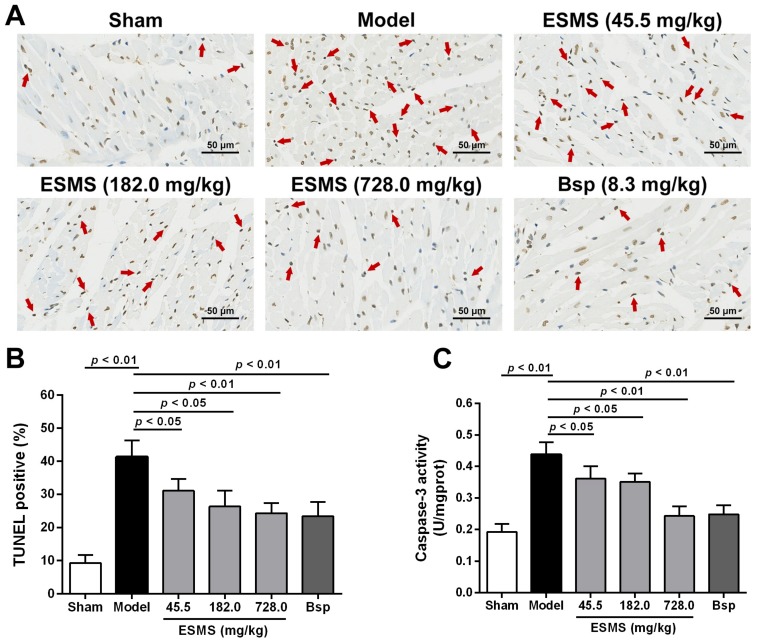
ESMS inhibited myocardial apoptosis in MI-induced HF mice. (**A**) Representative photomicrographs of terminal deoxynucleotidyl transferase-mediated deoxyuridine triphosphate nick-end labeling (TUNEL) staining images. The region of peri-infarcted was analyzed, and the arrow indicates apoptotic nuclei. Bar is 50 μm; (**B**) Quantitative analysis of apoptotic cells in TUNEL staining (*n* = 6); (**C**) Caspase-3 activity of all groups were determined in tissue after three weeks of CAL in mice (*n* = 8). Results were presented as mean ± SD. Bsp = Bisoprolol (positive control).

Myocardial apoptosis is an essential contributor to cardiac dysfunction after MI injury. The anti-apoptotic effects of ESMS were detected using the terminal deoxynucleotidyl transferase-mediated deoxyuridine triphosphate nick-end labeling (TUNEL) assay. As shown in Figure 3A,B, the number of TUNEL-positive cells (apoptotic cells) was significantly increased in the model group by comparison with that in the sham group. However, the apoptosis index was markedly decreased by ESMS (728.0 mg/kg) treatment compared with that in the model group. As shown in Figure 3C, MI injury increased caspase-3 activity, whereas ESMS significantly suppressed caspase-3 activity compared with group. Taken together, these findings suggest that ESMS has beneficial effects on cardiac function in mice with MI-induced HF.

### 2.5. ESMS Decreased Cardiomyocyte Injury and Apoptosis in H9c2 Cells Subjected to Oxygen Glucose Deprivation (OGD)

**Figure 4:**
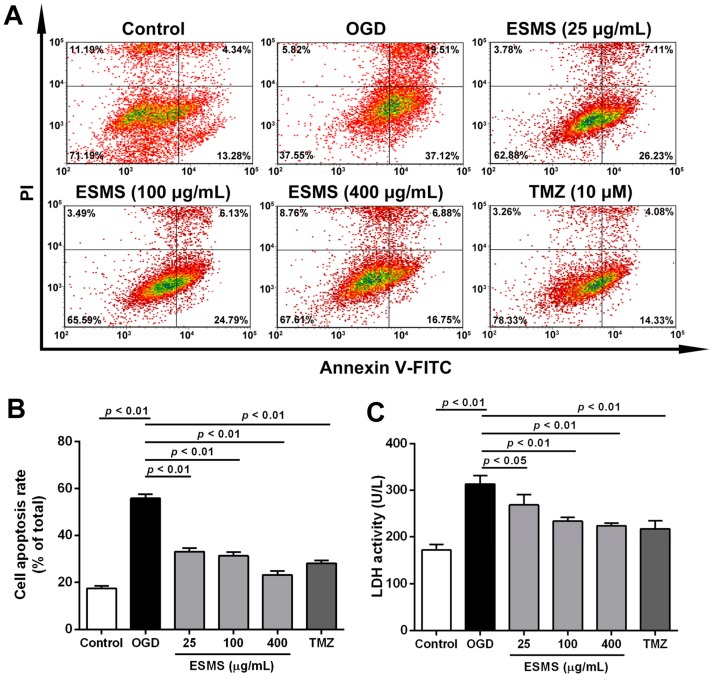
ESMS decreased apoptosis in H9c2 cells subjected to oxygen glucose deprivation (OGD). (**A**) Flow cytometric analysis of Annexin V-FITC/PI-stained H9c2 cells. Viable cells are Annexin V-FITC^−^ and PI^−^ early apoptotic cells are Annexin V-FITC^+^ and PI^−^ and late apoptotic cells are Annexin V-FITC^+^ and PI^+^ (*n* = 3); (**B**) The quantitative results were represented as the percentage of Annexin V-FITC^+^/PI^+^ cells among total cells (*n* = 3); (**C**) LDH release of all groups were determined (*n* = 6). Results were presented as mean ± SD. TMZ = Trimetazidine dihydrochloride (positive control).

The deleterious effects of different durations of OGD were investigated in H9c2 cells. The results showed that the viability of H9c2 cells decreased significantly by approximately 50% after 12 h of OGD (Figure S4A,B). Assessment of the viability of H9c2 cardiomyocytes showed that ESMS at concentrations of 25–800 μg/mL did not significantly affect cell viability (Figure S4C). OGD led to a decrease in H9c2 cell viability, whereas treatment with 25, 100, and 400 μg/mL ESMS maintained cell viability at 84.6 ± 3.0%, 89.7 ± 5.7%, and 95.1 ± 2.1%, respectively (Figure S4D). Quantitative analysis using flow cytometry confirmed that the H9c2 cell index was markedly increased compared with control group after OGD injury. However, ESMS treatment significantly decreased the apoptosis index (Figure 4A,B). Cardiomyocyte injury was assessed by determining the release of LDH, a marker of cell damage (Figure 4C). LDH release increased significantly in response to OGD compared with control group, whereas treatment with 25–400 μg/mL ESMS significantly reduced the LDH release.

### 2.6. ESMS Reduced Intracellular Ca^2+^ Levels, Increased ATPase Activity, and Decreased the Expression of Calcineurin A (CnA) in H9c2 Cells Subjected to OGD

**Figure 5:**
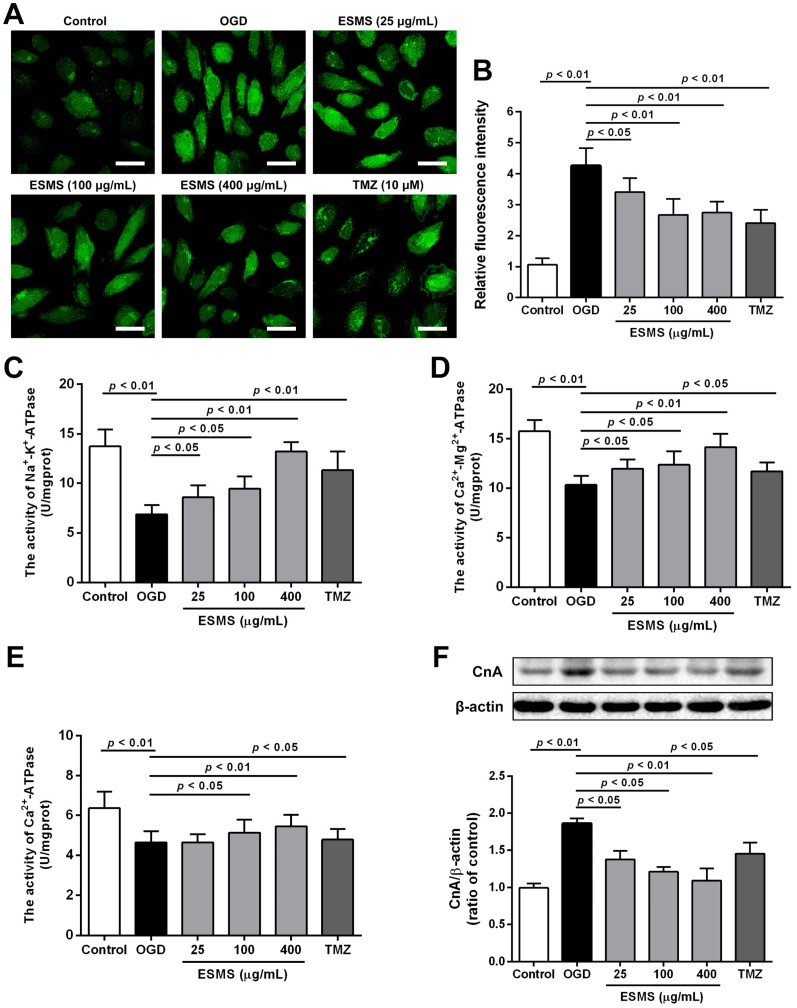
ESMS reduced intracellular Ca^2+^ levels, increased ATPase activity and reduced CnA protein expression in H9c2 cells subjected to OGD: (**A**) the intracellular Ca^2+^ levels were evaluated by probe Fluo-3 AM. Bar is 40 μm (*n* = 3); (**B**) quantitative analysis of intracellular Ca^2+^ levels (*n* = 3); Na^+^-K^+^-ATPase (**C**), Ca^2+^-Mg^2+^-ATPase (**D**), and Ca^2+^-ATPase (**E**) activities were determined (*n* = 6); and (**F**) CnA protein expression was detected by Western blotting (*n* = 3). Results were presented as mean ± SD. TMZ = Trimetazidine dihydrochloride (positive control).

As shown in Figure 5A,B, OGD injury significantly increased intracellular Ca^2+^ concentration, whereas ESMS significant reduced the intracellular Ca^2+^ levels. During OGD, Na^+^-K^+^-ATPase, Ca^2+^-Mg^2+^-ATPase and Ca^2+^-ATPase activities were significantly decreased compared with control group, while ESMS significantly increased ATPase activity. Previous studies showed that intracellular Ca^2+^ levels modulate the activity of calcineurin; therefore, we observed whether the expression of CnA was affected. Western blot analysis showed that CnA expression was increased in H9c2 cells subjected to OGD, whereas the ESMS (400 μg/mL) significantly inhibited increases in CnA expression.

### 2.7. ESMS Restored Mitochondrial Membrane Potential and Cellular ATP in H9c2 Cells Subjected to OGD

**Figure 6:**
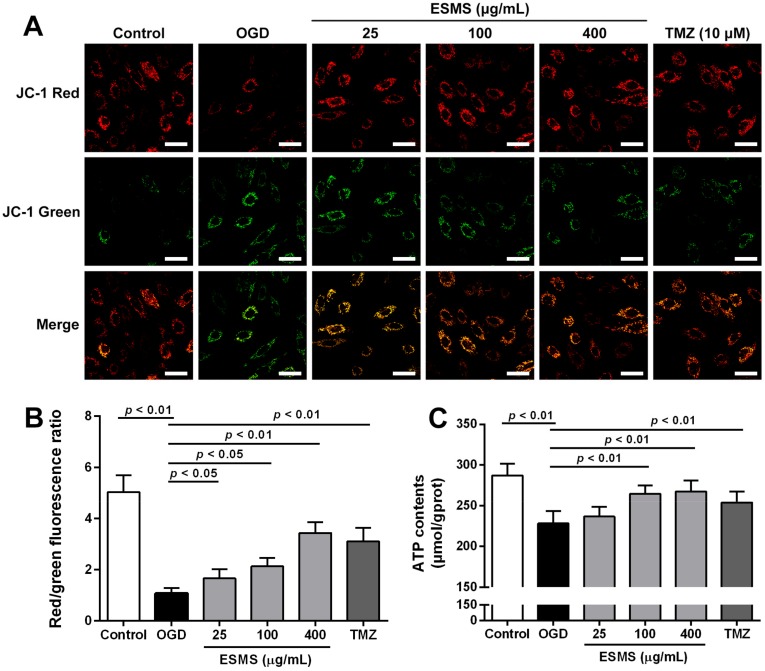
ESMS restored mitochondrial membrane potential and cellular ATP content in H9c2 cells subjected to OGD. (**A**) Mitochondrial membrane potential was evaluated by the probe JC-1. Green fluorescence shows JC-1 monomer, which enters in the cytosol following mitochondrial membrane depolarization. Red fluorescence indicates the JC-1 aggregation, which is accumulated in the inner membrane of mitochondria. Bar is 40 μm (*n* = 3); (**B**) Quantitative analysis of mitochondrial membrane potential (*n* = 3); (**C**) Cellular ATP content was evaluated (*n* = 6). Results were presented as mean ± SD. TMZ = Trimetazidine dihydrochloride (positive control).

Mitochondrial membrane potential was assessed using the JC-1 probe. As shown in Figure 6A,B, untreated cells exhibited bright-staining mitochondria that emitted red fluorescence. OGD caused the formation of monomeric JC-1, which indicated loss of membrane potential. ESMS treatment, however, decreased the OGD-induced formation of JC-1 monomers, suggesting that ESMS restored the OGD-induced loss of mitochondrial membrane potential. As shown in Figure 6C, OGD significantly depleted the cellular ATP content in H9c2 cells, whereas ESMS treatment largely abrogated the depletion of cellular ATP, which was favorable for cell survival.

### 2.8. ESMS Ameliorated the Expression of Mitochondrial Mediated Apoptosis Proteins and Reduced the Caspase-3 Activity in H9c2 Cells Subjected to OGD

**Figure 7:**
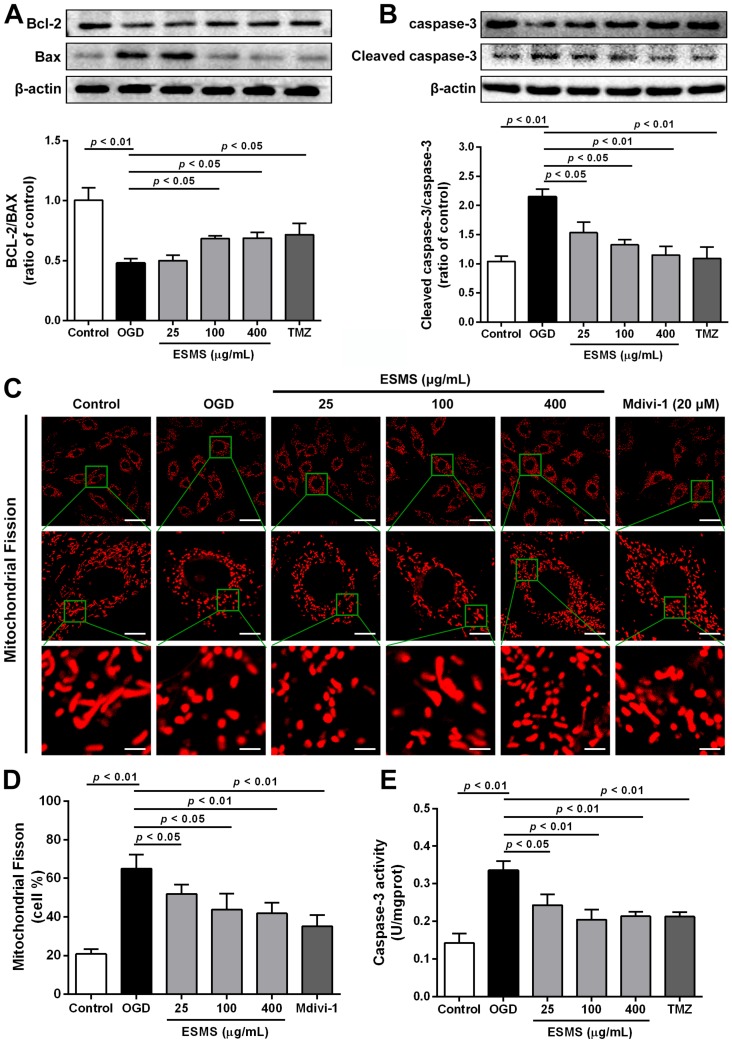
ESMS ameliorated the expression of mitochondrial mediated apoptosis proteins and reduced the caspase-3 activity in H9c2 cells subjected to OGD. (**A**) Bcl-2 and Bax expression were detected by Western blot (*n* = 3); (**B**) Caspase-3 protein expression was measured by Western blot (*n* = 3); (**C**) Mitochondrial fission was detected by Mito Tracker^®^ Deep Red FM with a confocal laser scanning microscope. Bars are 40 μm for top, 10 μm for middle and 4 μm for bottom (*n* = 3); (**D**) Quantitative analysis of mitochondrial fission (*n* = 3); (**E**) Caspase-3 activity of all groups was evaluated (*n* = 6). Results were presented as mean ± SD. TMZ = Trimetazidine dihydrochloride (positive control).

Bcl-2 and Bax play important roles in the regulation of cell apoptosis. As shown in Figure 7A, western blot analysis showed that OGD downregulated Bcl-2 and upregulated Bax, indicating the induction of apoptosis. However, the Bcl-2/Bax ratio was markedly increased in ESMS pretreated cells compared with that in the OGD group. As shown in Figure 7B,E, the expression of caspase-3 and activity were significantly increased in the OGD group by comparison with that in the control group, whereas treatment with ESMS significantly reduced the level of caspase-3. These results supported the anti-apoptotic effect of ESMS in H9c2 cells subjected to OGD.

### 2.9. ESMS Inhibited Mitochondrial Fission and Regulated Drp1 Phosphorylation and Translocation

**Figure 8:**
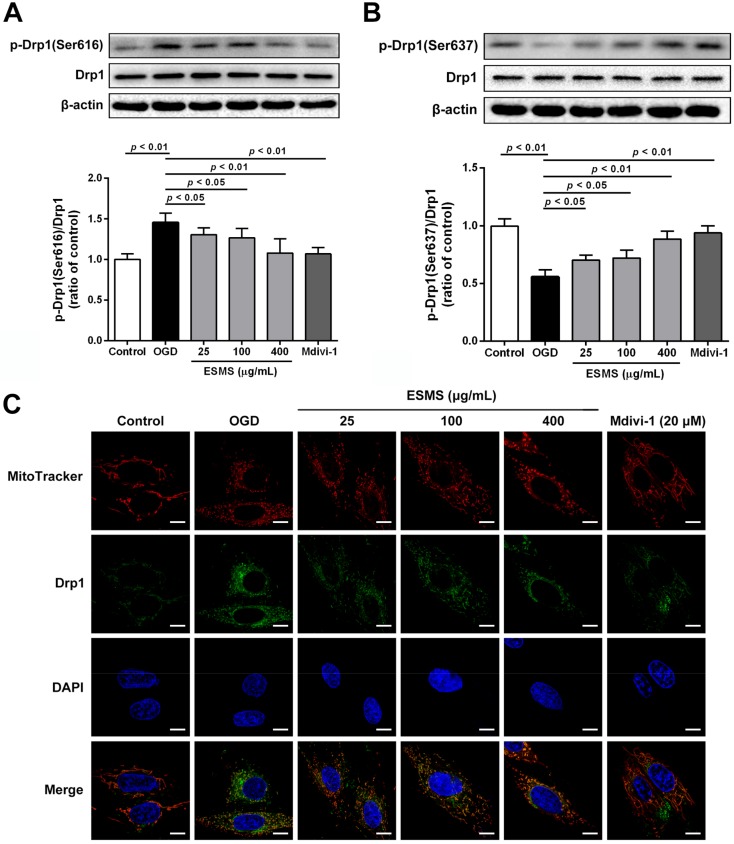
ESMS regulated Drp1 phosphorylation, translocation in H9c2 cells subjected to OGD. (**A**) Drp1 and p-Drp1 (Ser616) proteins expression levels were determined using Western blot analysis (*n* = 3); (**B**) Drp1 and p-Drp1 (Ser637) proteins expression levels were determined by Western blot analysis (*n* = 3); (**C**) Mitochondrial localization of Drp1 was assessed under confocal laser scanning microscope (green). Mitochondrial morphology was stained with Mito Tracker^®^ Deep Red FM (red). Nuclei were stained with 6-diamidino-2-phenylindole (DAPI) (blue). Differential interference contrast (DIC) images were also obtained. Bar is 10 μm (*n* = 3). Results were presented as mean ± SD.

Mitochondria show a flexible reticular formation in normal cells, whereas cells bearing predominantly fragmented or spherical mitochondria are considered to have undergone mitochondrial fission [22,34]. To investigate the changes in mitochondrial morphology, H9c2 cells were stained with Mito Tracker^®^ Deep Red FM (Life Technologies, Carlsbad, CA, USA). Representative images in Figure 7C,D show mitochondrial structures is elongated, branched, and interconnected in the control group. However, OGD injury increased mitochondrial fission, and small, round, and punctiform mitochondria became dominant. ESMS treatment significantly inhibited mitochondrial fission. To promote mitochondrial fission, Drp1 adjusts mitochondrial morphology, whose phosphorylation at Ser616 facilitates the mitochondrial fission while phosphorylation at Ser637 prevents it [24,25]. As shown in Figure 8A,B, no alterations in total Drp1 protein levels were observed during OGD injury; however, immunoblotting showed a remarkable increase in phosphorylation of Drp1 (p-Drp1) at Ser616 and a reduction in p-Drp1 at Ser637. ESMS inhibited Drp1 phosphorylation at Ser616 and increased Drp1 phosphorylation at Ser637, preventing mitochondrial fission associated with OGD injury. The translocation of Drp1 from the cytosol to mitochondria is an indicator of Drp1 activation [35]. The results showed that ESMS and the mitochondrial fission inhibitor Mdivi-1 had similar effects, inhibiting OGD injury-induced mitochondrial fission and reducing the amount of Drp1 in mitochondria in cardiomyocytes (Figure 8C).

## 3. Discussion

HF is an important and growing public health concern, and a leading cause of morbidity and mortality in industrialized countries worldwide [2]. MI has become the most important cause of HF. Moreover, the pathogenesis of MI injury-induced HF is a chronic and complex process that may involve abnormalities of energy metabolism, Ca^2+^ homeostasis, ventricular remodeling, altered expression or function of proteins, and defects in many organelles and cellular pathways [3]. Therefore, it is important to identify potential cardioprotective agents and elucidate their underlying mechanisms to prevent MI injury-induced HF.

The formula prescription SMS has been widely used for the treatment of cardiovascular diseases, with demonstrated clinical efficacy in the treatment of angina an HF in China [13,14,15]. Previous studies reported that SMS enhances myocardial systole, ameliorates left ventricular ejection fraction, regulates blood vessel function, modulates immunity, and has anti-lipid effects [11,18,19]. However, the molecular mechanism underlying the protective effect of SMS against MI-induced HF remains unclear. The results of the present study showed that ESMS ameliorated cardiac structure and function, modulated Ca^2+^-calcineurin-mediated Drp1 signaling pathways, regulated Ca^2+^ homeostasis, and inhibited mitochondrial-mediated apoptosis, thereby preventing further deterioration. These findings provide the first evidence of the protective mechanism of ESMS against myocardial injury during the progression from MI to HF.

Echocardiography is clinically utilized to evaluate cardiac structure and function [36,37]. Compared with other methods, echocardiography is economical and time saving, which is suitable for animal studies [38]. Another advantage of echocardiography is that it can determine the extent of myocardial remodeling and LV parameters [39]. In the present study, cardiac systolic function and LV parameters were assessed by echocardiography. The results showed that ESMS improved cardiac function, increasing LVEF and LVFS in mice with MI-induced HF.

The activities of certain cardiac marker enzymes reflect the pathological process of myocardial diseases. Myocardial enzymes such as LDH and CK are the most common biomarkers for MI and HF [19,40]. In addition, BNP increases as a result of left ventricular (LV) systolic dysfunction, and its quantification is recommended by the European Society of Cardiology guidelines as a test to rule out HF [41,42]. The results of the present study showed that MI (three weeks) significantly aggravated myocardial injury, as demonstrated by increased LDH, CK, and BNP release into the serum. However, treatment with different concentrations of ESMS significantly decreased LDH, CK activity, and BNP levels. These results strongly indicated that ESMS exerts a protective effect against MI-induced HF.

The loss of cardiomyocytes caused by apoptosis plays a leading role in various heart diseases, and, even worse, it unavoidably results in HF, as shown by accumulating evidence [43]. After ischemia, myocardial apoptosis is a significant pathogenic event. Inhibition of the apoptotic process prevents the loss of cardiomyocytes, minimizes cardiac injury, and delays or prevents the occurrence and development of MI injury [44]. In the present study, MI (three weeks) in vivo and OGD (12 h) in vitro significantly aggravated myocardial injury and decreased the cell viability, as well as increased cardiomyocyte apoptosis. Moreover, it also demonstrated a decrease in condensed and fragmented chromatin in the nuclei and an increase in the percentage of apoptotic cells. However, treatment with different concentrations of ESMS significantly decreased cell apoptosis. The anti-apoptotic effect of ESMS was shown to be useful in inhibiting cardiomyocyte apoptosis.

Based on the results that ESMS protected against cardiomyocyte apoptosis induced by OGD, we investigated the potential mechanisms underlying the effect of ESMS against MI injury. In cardiomyocytes, Ca^2+^ cycling plays a crucial role in myocyte contracting and relaxing. A series of specialized regulatory proteins precisely controls the intracellular Ca^2+^ homeostasis, including transcription factors, ion channels and Ca^2+^ binding proteins [45,46]. Moreover, calcium recycling is essential for cardiac function. Failure to recycle calcium results in overload and impaired cardiac function, ultimately leading to cell death [45]. Studies showed that targeting abnormal Ca^2+^ handling in HF may be beneficial. The current understanding of Ca^2+^ homeostasis during HF is largely focused on the end-stage of the disease, whereas little is known about changes in Ca^2+^ homeostasis during disease development. Existing data indicate that Ca^2+^ cycling is initially increased at early stages [47,48]. During MI, the intracellular Ca^2+^ concentration increases, resulting in acidosis; the decrease of Na^+^-K^+^-ATPase and Ca^2+^-ATPase activities result in impaired Ca^2+^ reuptake into the sarcoplasmic reticulum (SR), further increasing intracellular Ca^2+^ concentration [49]. The results of the present study showed that ESMS significantly increased Na^+^-K^+^-ATPase, Ca^2+^-Mg^2+^-ATPase, and Ca^2+^-ATPase activities and reduced intracellular Ca^2+^ concentration, thereby normalizing of intracellular Ca^2+^ levels.

Mitochondrial dynamics include fission and fusion processes that are controlled by critical regulatory proteins. Aberrant or increased fission leads to increased mitochondrial fragmentation and mitochondrial death or mitophagy [22]. However, several studies reported that preventing mitochondrial fission by inhibiting the fission protein Drp1 delays or inhibits apoptotic markers such as cytochrome c leakage and thereby cell death [21]. Drp1 localizes to the cytosol, and Drp1 activation is regulated by serine phosphorylation. Ser616 phosphorylation can facilitate activation of Drp1, which results in its translocation to the focal point of the future mitochondrial fission site, and leads to fission and follow-up mitochondrial dysfunction. However, Drp1 phosphorylation at Ser637 can inhibit activation, thereby preventing mitochondrial fission [24,25,33]. In the present study, OGD injury induced Drp1 activation, leading to mitochondrial fission. Western blot analysis and immunohistochemistry indicated that ESMS inhibited Drp1 phosphorylation at Ser616 and increased Drp1 phosphorylation at Ser637, preventing mitochondrial fission. The translocation of Drp1 from the cytosol to mitochondria is an indicator of Drp1 activation. ESMS inhibited OGD injury-induced mitochondrial fission and reduced the translocation of Drp1 at mitochondria in cardiomyocytes, thereby protecting mitochondrial functional and structural integrity.

Mitochondria provides at least 90% energy for cardiomyocytes, and serves as an important mediator in cardiomyocyte apoptotic signaling pathway [50]. In the present study, we confirmed that ESMS treatment restored mitochondrial membrane potential. Mitochondrial permeability and the release of the apoptosome are controlled by Bcl-2 family proteins. Bcl-2 and Bax are major proteins involved in the regulation of cell apoptosis [51,52]. The ratio of Bcl-2 to Bax determines survival or death following an apoptotic stimulus [53]. Western blotting analysis indicated that OGD injury induced Bax increased and Bcl-2 decreased, which favored cardiomyocyte apoptosis. However, ESMS inhibited the upregulation of Bax and downregulation of Bcl-2 (Figure 6A,B), suppressing cardiac myocyte apoptosis during OGD. To support our findings, we measured caspase-3 activity and expression. Our results indicated that ESMS downregulated caspase-3 expression and decreased its activity. Such an anti-apoptotic effect of ESMS is important for the reduction of OGD-induced cardiomyocyte dysfunction.

MI-disrupted Ca^2+^ leads to alterations of mitochondrial structure and function. Previous studies showed that cytoplasmic Ca^2+^ levels increase leading to activation of calcineurin, and sustained calcineurin activation is sufficient to damage cardiac structure and function [32]. In addition, calcineurin activation can increase mitochondrial fission through mechanisms involving the dephosphorylation of Drp1 [33]. Activated calcineurin dephosphorylates and activates Drp1, accelerating its translocation to mitochondria to stimulate mitochondrial fission [25,31,33].

As previously described, ESMS consists of bioactive compounds, some of which may protect against myocardial injury. Modern pharmacological studies illustrated that ginsenosides can be effective in improving ischemic myocardium metabolism, scavenging the free radicals, protecting the myocardial ultrastructure, and lessening the Ca^2+^ overload [54]. Ginsenosides Rb1 and Rb3 reduce cardiomyocyte apoptosis and oxidative stress induced by isoproterenol [18,55]. Ginsenosides Rg1 and Rb1 are effective in protecting cardiac hypertrophy associated with the activity of Ca^2+^-calcineurin signaling pathways [56,57]. Ginsenoside Re has a negative effect on cardiac contractility and autorhythmicity by altering cardiac electrophysiological properties [58,59]. In addition, Ophiopogonin D from *Ophiopogon japonicas* reduces intracellular Ca^2+^ overload and suppressed ER stress and stress-mediated apoptosis in cardiomyocytes [60]. Other important compounds in ESMS, Schizandrol A and Schisandrin B, protect against myocardial injury by acting against oxidative damage [18,61]. It has not been eliminated that it may work validly in a more complicated cascade associated with cardioprotection, which warrants further investigation. Therefore, further research and investigation are necessary to explore the protective role of ESMS in MI and HF via multiple mechanisms, such as anti-inflammatory, anti-apoptotic, anti-oxidative, and anti-autophagy effects.

## 4. Materials and Methods

### 4.1. Drugs and Reagents

The ESMS comprises of the ethanol extract of *Panax ginseng* C.A. MEY., water extract of *Ophiopogon japonicus* (Thunb.) KER-GAWL and *Schisandra chinensis* (Turcz.) BAILL at a ratio of 1:3:1.5. ESMS was provided from Tasly Pharmaceutical Co., Ltd. (Tianjin, China, batch number of 20150312), and its quality was confirmed by HPLC analysis (Figure S1). Trimetazidine dihydrochloride (TMZ), Bisoprolol (Bsp), and Mdivi-1 were purchased from Sigma (St. Louis, MO, USA). Fluo-3 AM, Probe JC-1, RIPA lysis buffer, and 4′, 6-diamidino-2-phenylindole (DAPI) were purchased from Beyotime (Shanghai, China). The FITC-Annexin V apoptosis detection kit was purchased from KeyGen Biotech. Co., Ltd. (Nanjing, China). Mito Tracker® Deep Red FM and was obtained from Life Technologies (California, CA, USA). Dulbecco’s modified Eagle’s medium (DMEM) was purchased from GIBCO/BRL (California, CA, USA). Fetal bovine serum (FBS) was obtained from ScienCell (Carlsbad, CA, USA).

### 4.2. Animals

Male ICR mice weighing 18–25 g were purchased from the Model Animal Research Centre of Yangzhou University (Yangzhou, China, certificate number SCXK 2012-0004). All procedures and assessments were approved by the Animal Ethics Committee of the School of Chinese Materia Medica, China Pharmaceutical University (Approval No.: 2121961; 20 April 2016). The experiments were carried out in accordance with the National Institutes of Health Guide for the care and use of laboratory animals (NIH Publication number 80-23, revised 1996). Mice were allowed a 1-week acclimatization period prior to entry into any experimental protocol. Prior to experiments, all animals were randomized into experimental groups and measured blindly.

### 4.3. Surgical Preparation

The model was produced as previously described [38]. The mice were anesthetized with chloral hydrate (400 mg/kg, i.p.). Following tracheal intubation, aerobic positive pressure ventilation was performed. Anterior thoracotomy was performed to open the pericardium under sterile conditions. Consequently, the heart was exposed, and the left anterior descending (LAD) coronary artery was rapidly ligated approximately 3–4 mm distal from its origin using of a 6-0 silk suture. Sham-operated animals were subjected to a similar surgical procedure without LAD ligation.

The mice were randomized into six groups: sham, myocardial ischemia model, Bisoprolol, and three ESMS groups treated with different doses. According to the protocol, the treatment groups were orally administered ESMS (45.5, 182.0, and 728.0 mg/kg) or Bisoprolol (8.3 mg/kg). The sham and model groups were treated with the same volume of double distilled water. The treatment was maintained for three weeks (once a day).

### 4.4. Echocardiographic Analysis

After treatment, mice underwent an echocardiographic by using a Vevo 2100 Micro-imaging System (Visual Sonics, Toronto, ON, Canada) with a 30-MHz probe. The mice were anesthetized using 2.5% isoflurane. Each mouse was transferred to dorsal recumbency and then was placed on a heated imaging platform after fully anesthetized. The following parameters were calculated: LVEF, LVFS, IVS; d, LVPW; d, LVID; d, LV Vol; d, LV Mass, and RWT. All measurements were averaged for three consecutive cardiac cycles by an experienced technician who was the study groupings.

### 4.5. Histologic Examination

At the end of the experiment, the heart tissue was removed, fixed with 4% paraformaldehyde, embedded in paraffin, sliced into sections of 5 μm thickness, stained with hematoxylin–eosin and Masson’s trichrome, and mounted. The histopathological changes were observed with a light microscope (Leica, Wetzlar, Germany).

### 4.6. Evaluation of LDH, CK Activities and BNP Levels in Serum

At the end of the experiment, blood samples were collected. Serum samples were obtained by centrifugation of the blood samples at 15 °C for 10 min at 3500 rpm. LDH and CK activities were determined using respective kits (Nanjing Jian Cheng Biotech Co., Ltd., Nanjing, China), and BNP was measured using an ELISA kits (Nanjing Jian Cheng Biotech Co., Ltd.), according to the manufacturer’s instructions.

### 4.7. Measurement of ATPase Activities, Ca^2+^ Levels, and Caspase-3 Activity in Heart Tissues

At the end of the experiment, the hearts were removed and gently washed in 0.9% sodium chloride. LV samples were taken, weighed and homogenized in lysis buffer and centrifuged at 4 °C for 10 min at 3500 rpm. The Na^+^-K^+^-ATPase, Ca^2+^-Mg^2+^-ATPase, Ca^2+^-ATPase activities, and Ca^2+^ levels were determined using the respective kits (Nanjing Jian Cheng Biotech Co., Ltd.), following the manufacturer’s instructions. Heart tissue samples were weighed, homogenized in lysis buffer, and centrifuged at 4 °C for 10 min at 16,000× *g*. Caspase-3 activity was determined using a caspase-3 activity assay kit (Beyotime, Shanghai, China), following the manufacturer’s instructions.

### 4.8. TUNEL Staining for Apoptosis In Vivo

The myocardium was harvested and fixed in 4% paraformaldehyde solution at three weeks after treatment. TUNEL staining was performed using the In Situ Cell Death Detection Kit, POD (Roche Diagnostics, Indianapolis, IN, USA) following the manufacturer’s instructions. For each slice, five fields were randomly obtained under a light microscope (Leica). The region of peri-infarcted was analyzed, and the extent of cell apoptosis was expressed as the ratio of TUNEL positive nuclei.

### 4.9. Cell Culture

The H9c2 cardiomyocyte cell line was obtained from Shanghai Institute of Cell Biology, Chinese Academy of Sciences (Shanghai, China). H9c2 cells were maintained in DMEM (supplemented with 10% FBS, 100 U/mL penicillin and 100 μg/mL streptomycin), and exposure to a humidified atmosphere of 5% CO_2_ and 95% air at 37 °C. The cells were grown to 80–90% confluence prior to use in experiments.

### 4.10. Oxygen-Glucose Deprivation (OGD) and Drug Treatment

The H9c2 cell cultures were subjected to OGD for 12 h by incubation in a deoxygenated glucose-free balanced salt solution. The OGD injury was produced by incubation with none-glucose DMEM and exposure to a hypoxic environment of 94% N_2_, 5% CO_2_, and 1% O_2_ at 37 °C. There were six different groups: control, OGD exposure (model), various concentrations of ESMS (25, 100, and 400 μg/mL), and TMZ (10 μM) as a positive control for 12 h during OGD treatment. ESMS was dissolved in none-glucose DMEM, and TMZ was dissolved in phosphate buffer saline (PBS). Mitochondrial division inhibitor (Mdivi-1) was dissolved in dimethyl sulfoxide (DMSO, SunShineBio, Nanjing, China), which was diluted with none-glucose DMEM to ensure the final concentration of DMSO was 1:10,000.

### 4.11. Cell Viability and LDH Assays

Cell viability was determined through 3-(4,5-Dimethylthiazol-2-yl)-2,5-diphenyltetrazolium bromide (MTT) assay. Cells were seeded at 10,000 cells/well in 96-well plates. After different treatments, cells were incubated with MTT at a final concentration of 0.5 mg/mL for another 4 h. Then, the medium was discarded, 150 μL of DMSO was added with shaking for 10 min to dissolve the formazan crystals. The absorbance was read measured using a microplate reader (Epoch, BioTek, Norcross, GA, USA) at a detection wavelength of 570 nm, with a reference wavelength of 650 nm, and cell viability was expressed as a percentage of the untreated control values. After different treatments, the culture supernatants were collected. The release of LDH was tested to further measure the extent of cell injury. The activity of LDH was determined using a LDH activity assay kit, following the manufacturer’s instructions.

### 4.12. Flow-Cytometric Analysis for Apoptosis

After each treatment, H9c2 cells were collected, washed with cold PBS, resuspended in Annexin V-FITC binding buffer. Then, the cells were incubated with Annexin V-FITC/PI for 5 min in the dark. Cellular fluorescence was analyzed by a flow cytometric assay using MACSQuant Analyzer 10 (Miltenyi Biotec, Bergisch Gladbach, Germany). At least 10,000 cells were detected in all experiments, and the data were analyzed with FCS Express V3 (DeNovo, Los Angeles, CA, USA).

### 4.13. Measurement of ATPase Activities, ATP Content and Caspase-3 Activity In Vitro

H9c2 cardiomyocytes were lysed in ice-cold lysis buffer and centrifuged at 4 °C for 10 min at 3500 rpm. The Na^+^-K^+^-ATPase, Ca^2+^-Mg^2+^-ATPase, and Ca^2+^-ATPase activities were determined using the respective kit following the manufacturer’s instructions. For assessment of caspase activity, cardiomyocytes were lysed in ice-cold lysis buffer and centrifuged at 4 °C for 10 min at 16,000× *g*. Caspase-3 activity was determined using a caspase-3 activity assay kit, following the manufacturer’s instructions.

### 4.14. Measurement of Intracellular Ca^2+^ Concentration 

H9c2 cells were seeded at a density of 10,000 cells/dish in 35-mm confocal dishes (Glass Bottom Dish, Bratislava, Slovakia). After each treatment, the cells were washed three times with PBS and then incubated with Fluo-3 AM (10 μM) for 30 min at 37 °C. Then, the solution was discarded, extracellular unloaded free Fluo-3/AM was completely washed off with PBS, and images were scanned with a confocal laser scanning microscope (LSM700, Zeiss, Jena, Germany). Fluorescence intensity was measured. (Fluo-3/AM: excitation/emission = 488/525 nm).

### 4.15. Measurement of Mitochondria Membrane Potential

H9c2 cells were seeded at a density of 10,000 cells/dish in 35-mm confocal dishes (Glass Bottom Dish). After each treatment, the cells were incubated with JC-1 staining solution (10 μg/mL) for 25 min at 37 °C. The cells were washed three times with PBS and immediately analyzed with a confocal laser scanning microscope. Green fluorescence reflects JC-1 monomers, which enters into the cytosol following mitochondrial membrane depolarization. Red fluorescence is ejected from JC-1 aggregation in the inner membrane of mitochondria. Data were expressed as the ratio of red to green fluorescence intensity, which indicates the level of depolarization of the mitochondrial membrane (JC-1 aggregates: excitation/emission = 543/590 nm; JC-1 monomers: excitation/emission = 488/525 nm).

### 4.16. Mitochondrial Fission Analysis

H9c2 cells were seeded at a density of 10,000 cells/dish in 35-mm confocal dishes (Glass Bottom Dish, Bratislava, Slovakia), and treated with ESMS and Mdivi-1 (20 μM) with the OGD injury. After each treatment, the cells were washed with PBS. Then they were incubated at 37 °C with Mito Tracker® Deep Red FM (400 nM) for 30 min. The cells were followed up with fixing, permeabilizing, and incubation with anti-Drp1 primary antibody. The cells were subsequently incubated with an Alexa Fluor 488 conjugated Donkey Anti-Goat IgG (H+L) antibody and DAPI. The structures of mitochondria were viewed with a confocal laser scanning microscope. (Mito Tracker^®^ Deep Red FM: excitation/emission = 644/665 nm; Alexa Fluor 488 conjugated Donkey Anti-Goat IgG (H+L) antibody: excitation/emission = 493/519 nm; DAPI: excitation/emission = 340/488 nm).

### 4.17. Western Blotting Analysis

H9c2 cells were lysed with RIPA lysis buffer that was supplemented with protease inhibitors. The whole cell lysates were centrifuged at 4 °C for 10 min at 12,000 rpm, and protein concentration was quantified using the BCA Protein Assay kit (Beyotime, Shanghai, China). Equal amounts of proteins (35 μg) were separated by 10% SDS-PAGE, and subsequently transferred to polyvinyl difluoride (PVDF) membranes (Millipore Corporation, Bedford, MA, USA) by electrophoresis. The membranes were blocked with 3% BSA and stained with primary antibodies against Bax (1:1000), Bcl-2 (1:1000) and caspase-3 (1:1000), all from CST (Boston, MA, USA); CnA (1:5000, Abcam, Cambridge, MA, USA); Drp1 (1:1000, CST); p-Drp1(Ser637) (1:1000, CST); p-Drp1(Ser616) (1:1000, CST); and β-actin (1:1000, CST) for 16 h at 4 °C. The primary antibodies were followed by incubation with the HRP-conjugated secondary antibodies (1:8000, Bioworld, Louis Park, MN, USA). The visualization of protein signal was detected using enhanced chemiluminescence (ECL, Vazyme Biotech, Nanjing, China). The results were quantified using Image Lab™ software (version 4.1, Bio-Rad, Hercules, CA, USA).

### 4.18. Statistical Analysis

All data were presented as the means ± SD. One-way analysis of variance (ANOVA) was performed to analyze the data, followed by Dunnett’s post-hoc test when the data involved three or more groups. Values of *p* < 0.05 were considered statistically significant. In addition, all statistical analyses were performed using GraphPad Prism 6.01 (GraphPad Software, Inc., La Jolla, CA, USA).

## 5. Conclusions

Our findings indicated for the first time that ESMS can attenuate MI-induced HF by improving cardiac function, repairing cardiac structural damage, and reducing histopathological damage. ESMS attenuated MI injury-induced HF by modulating Ca^2+^-calcineurin-mediated Drp1 signaling pathways, thereby inhibiting mitochondrial mediated apoptosis. These findings provided insight into the function and mechanism of ESMS in the treatment MI-induced HF.

## Supplementary Materials

Supplementary materials can be found at www.mdpi.com/1422-0067/18/9/1825/s1.

## Abbreviations

HF	Heart failure
MI	Myocardial ischemia
ESMS	Extract of Sheng-Mai-San
OGD	Oxygen-glucose deprivation
LVEF	Left ventricular ejection fraction
LVFS	Left ventricular fractional shortening
LDH	Lactate dehydrogenase
CK	Creatine kinase
BNP	Brain natriuretic peptide
CnA	Calcineurin A
Drp1	Dynamin-related protein 1
Bcl-2	B cell lymphoma/lewkmia-2
BAX	Bcl-2 Assaciated X protein
Caspase-3	Cysteinyl aspartate specific proteinase-3
