# Supplementary material for: Extract of Sheng-Mai-San Ameliorates Myocardial Ischemia-Induced Heart Failure by Modulating Ca2+-Calcineurin-Mediated Drp1 Signaling Pathways
Source: Int J Mol Sci. 2017 Aug 25;18(9):1825. doi: 10.3390/ijms18091825 (PMC5618477; doi:10.3390/ijms18091825)
Supplement: Supplementary file 1 [file ijms-18-01825-s001.pdf]

Supplementary information for

Extract of Sheng-Mai-San Ameliorates Myocardial Ischemia-Induced Heart Failure by Modulating Ca^{2+}-Calcineurin-Mediated Drp1 Signaling Pathways

Ye Yang [1], Yushan Tian [1], Siyao Hu [1], Suxia Bi [1], Suxia Li [1], Yuanjia Hu [2], Junping Kou [1], Jin Qi [1,*] and Boyang Yu [1,*]

1 State Key Laboratory of Natural Medicines, Jiangsu Key Laboratory of Traditional Chinese Medicine Evaluation and Translational Research, Department of Complex Prescription of Traditional Chinese Medicine, China Pharmaceutical University, Nanjing 211198, China; yangye459@163.com (Y.Y.); yushan126163@126.com (Y.T.); hu_siyao@126.com (S.H.); suxiabi@163.com (S.B.); lsx0601@126.com (S.L.); junpingkou@cpu.edu.cn (J.K.)

2 State Key Laboratory of Quality Research in Chinese Medicine, Institute of Chinese Medical Sciences, University of Macau, Macau 999078, China; yuanjiahu@umac.mo (Y. H.)

* Correspondence: 1020060886@cpu.edu.cn (J.Q.); boyangyu59@163.com (B.Y.); Tel./Fax: +86-25-8618-5158 (J.Q.&B.Y.)

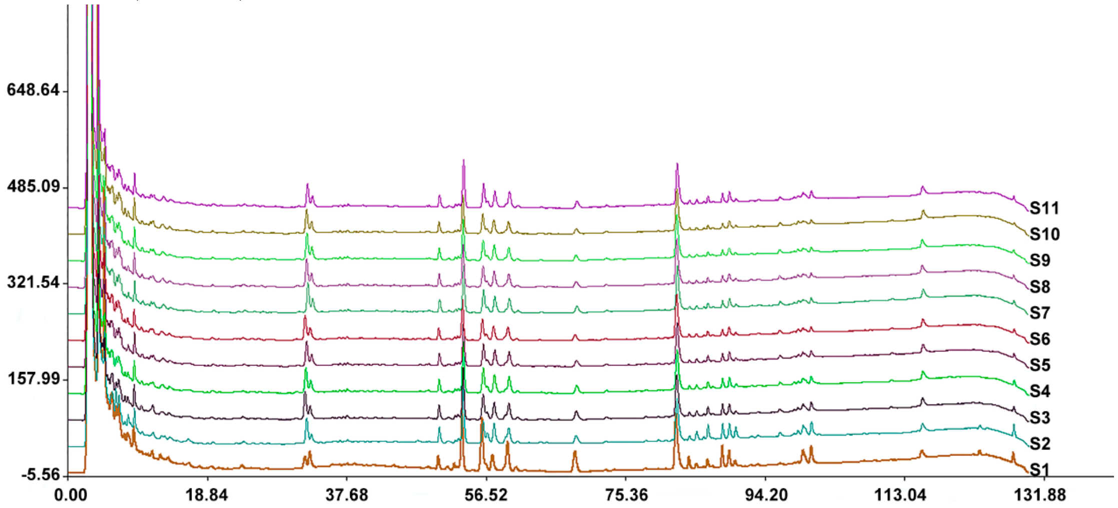

Figure S1. HPLC-DAD chromatographic fingerprints of 11 batches ESMS samples chromatogram at 203 nm. The chromatographic separation was operated on an Kromasil 100-5C18 (250 mm × 4.6 mm, i.d. 5μm) maintained at 30℃. The mobile phase consisted of 0.01% formic acid in acetonitrile (solvent A) and 0.01% formic acid in water (solvent B). The gradient program as follows: 20% A for 0 min, 20% A for 15 min, A for 25 min, 32% A for 45 min, 34% A for 65 min, 42% A for 75 min, 60% A for 95 min, A for 110 min, 100% A for 125 min, A for 130 min. The batches number of ESMS samples: 20150101 (S1), 20150301 (S2), 20150305 (S3), 20150306 (S4), 20150307 (S5), 20150308 (S6), 20150312 (S7), 20150313 (S8), 20150314 (S9), 20150408 (S10), 20150409 (S11).

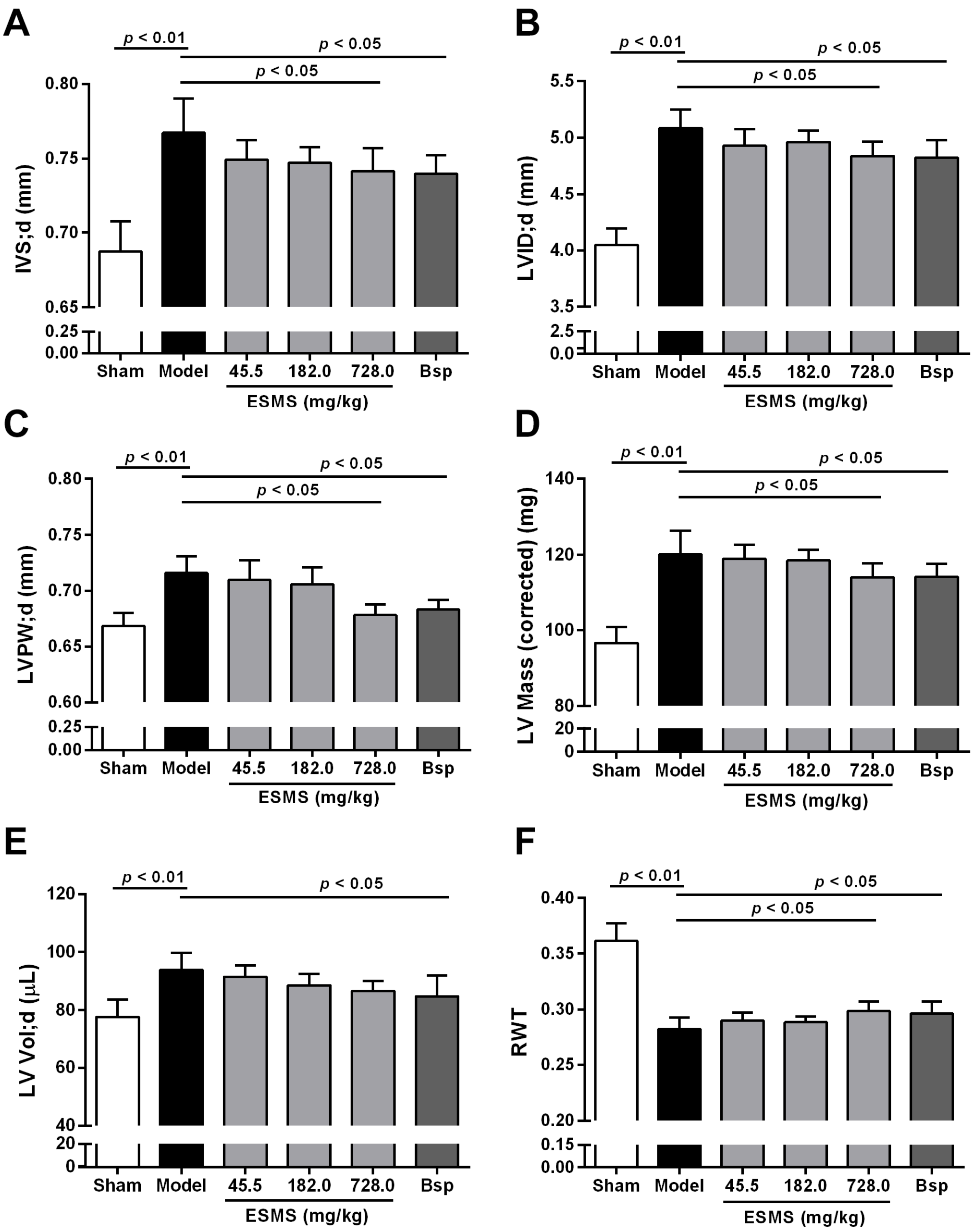

Figure S2. ESMS improved the cardiac structure parameters in MI-induced HF mice. Cardiac structure parameters IVS; d (A), LVID; d (B), LVPW; d (C), LV Mass (D), LV Vol; d (E), RWT (F) were determined by echocardiography after 3 weeks of CAL in mice. (n = 8). Results were presented as mean ± SD. Bsp = Bisoprolol (positive control).

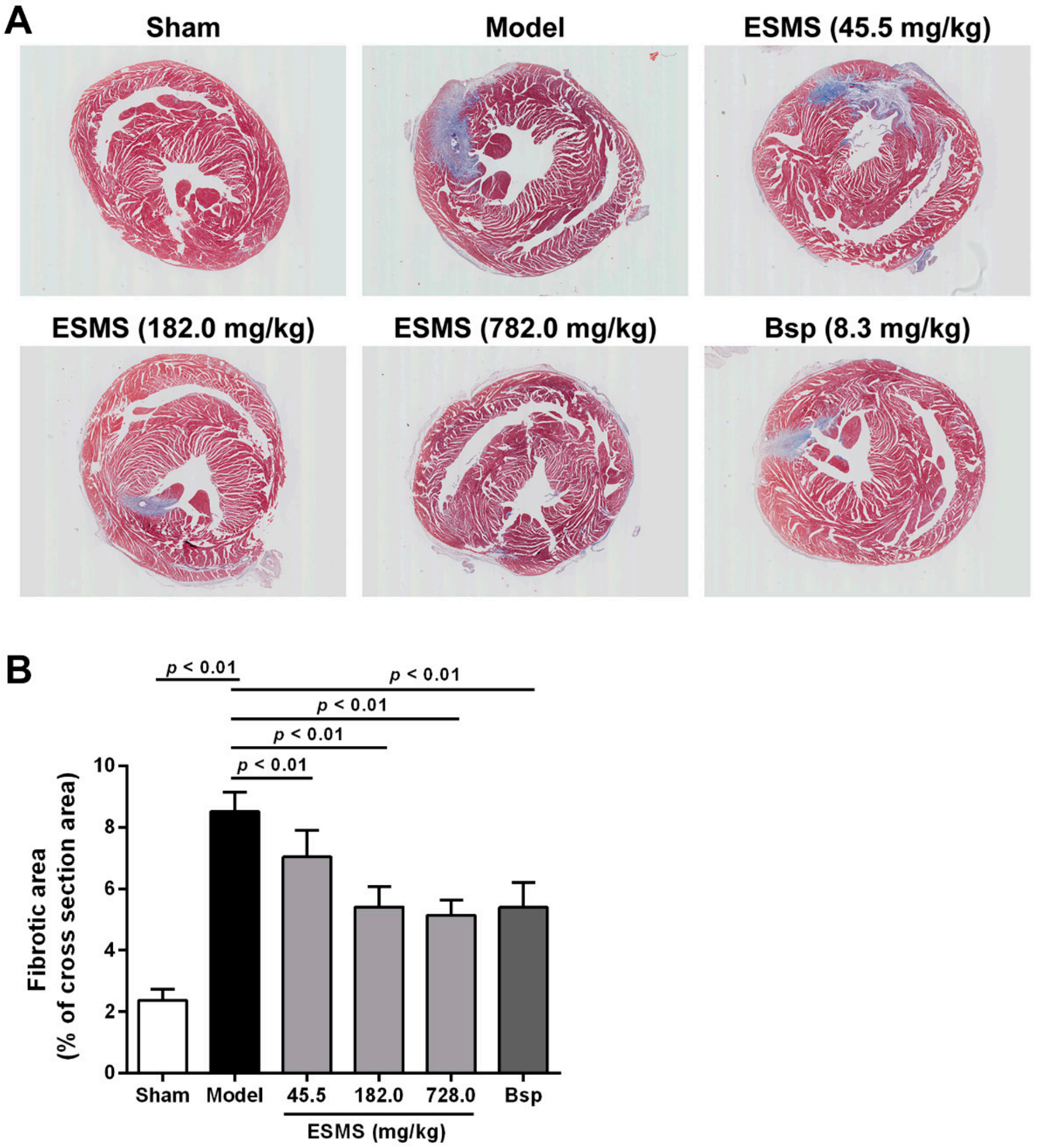

Figure S3. ESMS decreased myocardial fibrosis in MI-induced HF mice. (A) Representative scanned images of myocardial fibrosis using Masson's trichrome staining. (B) The quantification of fibrotic area was analyzed by Image Por Plus 6.0 (Media Cybernetics, Silver Spring, USA) (n = 6). Results were presented as mean ± SD. Bsp = Bisoprolol (positive control).

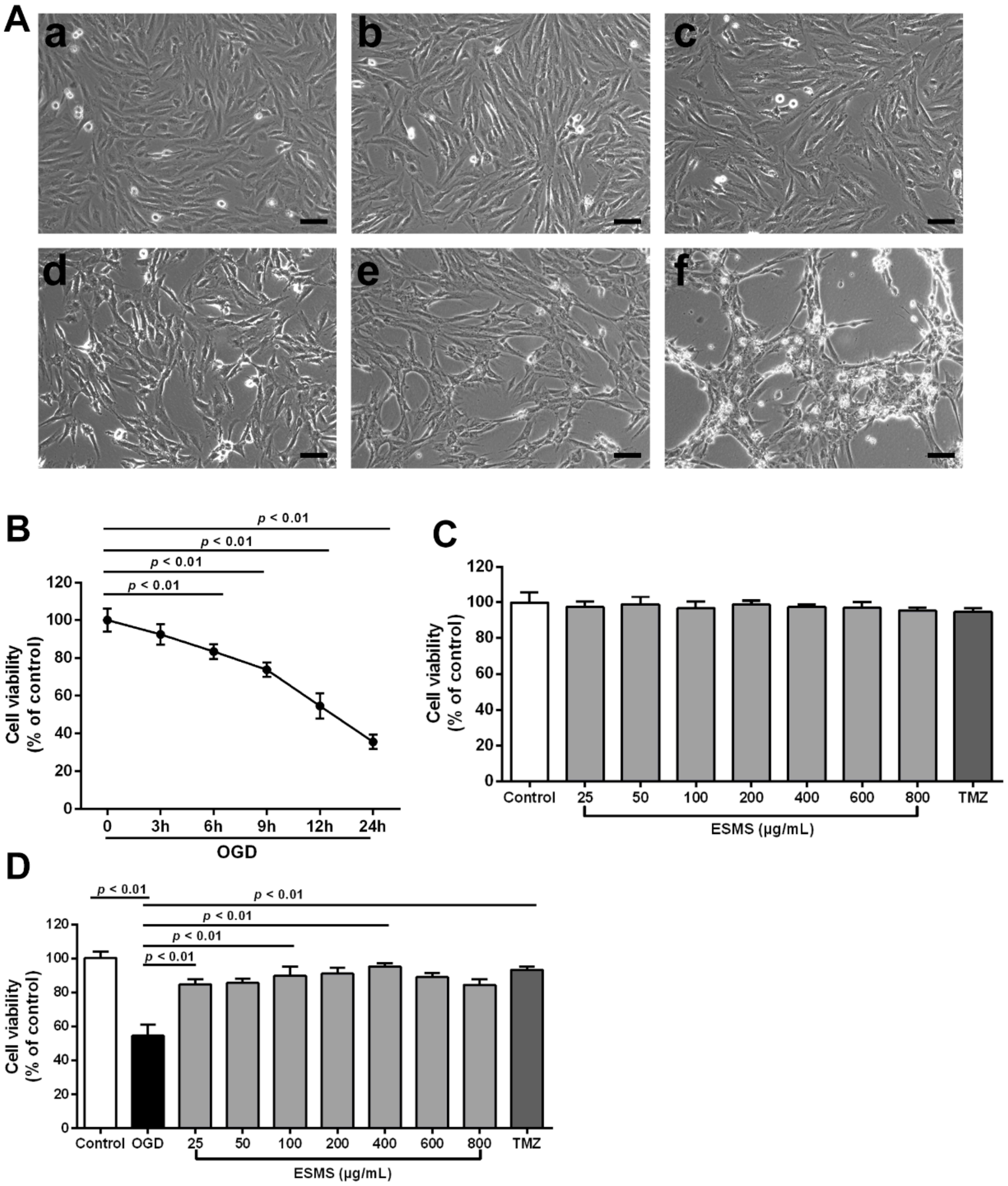

Figure S4. ESMS decreased cardiomyocytes injury. (A) H9c2 cells morphology with OGD. (a) control; (b) 3 h, (c) 6 h, (d) 9 h, (e) 12 h, (f) 24 h. Bar is 20 μm. (B) Time course of the viability of H9c2 cells induced by OGD. Cell viability was measured by the MTT assay described in method after 0, 3, 6, 9, 12, 24 h OGD (n = 6). (C) H9c2 cells were pretreated with ESMS at the concentration of 25, 50, 100, 200, 400, 800 μg/mL for 24 h. Cell viability was measured by the MTT assay described in method (n = 6). (D) H9c2 cells were treated with ESMS (25-800 μg/mL) and then exposed to OGD for 12 h. The cell viability was detected using MTT assay described in method (n = 6). Results were presented as mean ± SD. TMZ = Trimetazidine dihydrochloride (positive control).
